# Serum osteoinductive factor is associated with microalbuminuria and diabetic nephropathy in type 2 diabetes

**DOI:** 10.1097/MD.0000000000011759

**Published:** 2018-08-03

**Authors:** Wen Wei, Mei Tu, Rong Huang, Tong Chen

**Affiliations:** Department of Endocrinology, The First Hospital of Longyan Affiliated to Fujian Medical University, Longyan, Fujian, China.

**Keywords:** diabetic nephropathy, osteoinductive factor, predictor, serum

## Abstract

We investigated the relationship between serum osteoinductive factor (OIF) and diabetic nephropathy (DN), and its potential use as a diagnostic marker for DN.

This study included 177 patients with type 2 diabetes mellitus (T2DM) with normoalbuminuria, 42 with DN and microalbuminuria, and 49 with DN and macroalbuminuria, as well as 296 controls. Baseline characteristics, microalbuminuria prevalence, macroalbuminuria prevalence, and diabetic complications were compared between OIF quartiles. Serum OIF was examined by enzyme-linked immunosorbent assay. Other clinical parameters were measured using standard methods. Correlations between OIF and clinical parameters were assessed using Pearson correlation. Predictive value of OIF for DN was assessed using multivariate logistic regression. Receiver operator characteristic (ROC) curves were used to identify the optimal sensitivity for serum OIF.

Univariate analysis showed microalbuminuria prevalence negatively correlated with OIF, 4.3% for quartile 1 (Q1) (>367.5 pg/mL), 13.7% for Q2 (320.3–367.5 pg/mL), 17.9% for Q3 (275.0–320.3 pg/mL), and 28.8% for Q4 (<275.0 pg/mL) (*P*_trend_ < .001), as did T2DM complications. ROC analysis showed an OIF of <343.4 pg/mL was predictive of DN (C statistic 0.702). OIF <343.4 pg/mL remained predictive of microalbuminuria (odds ratio = 11.60; 95% confidence interval: 1.25–107.47) after adjusting for confounding factors.

Serum OIF is an independent diagnostic marker of DN.

## 1 Introduction

Type 2 diabetes mellitus (T2DM) has emerged over recent decades as a major health problem that is now estimated to affect more than 300 million people worldwide.^[1]^ Diabetic nephropathy (DN) is a serious and progressive microvascular complication of T2DM, characterized by persistent albuminuria, arterial blood pressure elevation, and a decline in glomerular filtration rate (GFR).^[2]^ DN has now become the leading cause of end-stage renal disease and is a key cause of diabetes-related disability and death worldwide.^[3]^ Due to the irreversible progressive development of DN, its large impact on disease prognosis, and patient's quality of life, early diagnosis and treatment is clinically significant. Microalbuminuria is considered to be an early marker of DN in clinical practice. However, emerging evidence suggests that glomerular damage is also present in diabetes patients with normoalbuminuria,^[4,5]^ who may be missed if diagnosis is based only on the detection of microalbuminuria. There is an urgent need to determine potential risk factors for early DN onset and progression.

Recent research indicates that osteoinductive factor (OIF) may have a role in the pathogenesis of DN, and that serum OIF could be a potential diagnostic marker for detecting the early stages of DN.^[6]^ OIF is a secreted glycoprotein first isolated from bovine bone matrix. Due to its growth stimulating activity through the actions of bone morphogenetic protein (BMP)-2 and BMP-3, this protein is also named osteoglycin.^[7]^ OIF has a tissue-specific glycosylation site and different post-translational modifications in different tissues. The OIF gene is widely expressed in the sclera, cornea, lung, skeletal muscle, testis, bone, adrenal gland, pituitary gland, heart, and blood vessels.^[8,9]^ The human OIF gene is located at 9q22, with 92% and 85% sequence homology with cow and rat, respectively.^[10]^ The high conservation among species indicates the important physiological functions of OIF. These include the inhibition of osteoclast and osteoclast-like cells, the induction of heterotopic bone, a role in arthrodesia, possible constituent roles in the vascular matrix,^[11]^ and possible roles in the proliferation of tumor cells.^[12]^ OIF co-expresses with adrenocorticotropic hormone (ACTH) in the pituitary, and interacts with ACTH, maintaining the balance of the hypothalamic–pituitary–adrenal axis functions.^[13]^ Recent research has revealed that OIF is involved in the development of angiogenesis and atherosclerosis. Vascular endothelial injury and atherosclerosis of renal arteries is an important mechanism for the pathogenesis and development of DN. However, there has been little investigation of the relationship between OIF and DN.

The aim of the present study was to investigate further the correlation between serum OIF and DN, to investigate the possible mechanism linking OIF and DN, and to assess the value of serum OIF in the early diagnosis and monitoring of DN. We hypothesized that serum OIF is involved in the pathogenesis and development of DN and can be used as a diagnostic marker of DN.

## 2 Materials and methods

### 2.1 Subjects

A cross-sectional study was carried out between July 2014 and June 2015 based at the First Hospital of Longyan Affiliated to Fujian Medical University. A total of 268 male patients were recruited for the study aged 20 to 70 years with T2DM with normoalbuminuria, DN with microalbuminuria (urinary albumin excretion rate [UAER] 20–200 μg/min), and DN with macroalbuminuria (UAER >200 μg/min) according to the American Diabetic Association criteria.^[14]^ Patients were excluded from the study if they were suffering from acute metabolic disturbance including ketoacidosis, hyperglycemia, hyperosmolar status, acute severe infection, other chronic or acute renal diseases were on hemodialysis, or had a bone fracture within the previous 3 months, autoimmune disease, malignant cancer, severe cardiac insufficiency, coronary heart disease (CHD), liver disease, or acute cerebral infarction. In addition, 296 age and sex-matched individuals in the Health Examination Center during the same period were selected as controls. Potential control subjects with diabetes, liver disease, renal disease, cerebrovascular disease, autoimmune disease, or malignant tumor were excluded from the study. The study was approved by the Ethics Committee of the First Hospital of Longyan Affiliated to Fujian Medical University. Informed consent was obtained from each patient and control subject for participation in the study and collection of urine and blood samples.

### 2.2 Clinical data and laboratory tests

The medical history (duration of diabetes and hypertension), height, weight, and brachial arterial pressure, measured by desktop mercurial sphygmomanometer in the patient's resting state, were recorded for each participant. Venous blood was sampled after 12 hours of fasting for all subjects. Fasting blood samples were used to determine fasting blood glucose, fasting C-peptide, glycated hemoglobin (HbA1c), triglyceride (TG), total cholesterol (TC), high-density lipoprotein cholesterol (HDL-C), low-density lipoprotein cholesterol (LDL-C), apolipoprotein A, apolipoprotein B, lipoprotein(a) (LP(a)), serum creatinine (SCr), cystatin C (CYSC), homocysteine (HCY), and C-reactive protein using an Olympus Automated Chemistry Analyzer AU2700 (Beckman Coulter, Brea, CA). Patients provided a 24-hour urine sample collected between 7:00 am and 7:00 am the following day. Urine was used to measure the concentration of microalbuminuria by rate scatter nephelometry in a BNII ProSpec and to compute UAER after the correction of urine volume per unit time. Estimated glomerular filtration rate (eGFR) was calculated by applying the CKD-EPI formula to the SCr concentration.

### 2.3 Measurement of serum OIF

Serum OIF concentration was determined using an enzyme-linked immunosorbent assay (ELISA) kit following the manufacturer's protocols (Shanghai Xin Yu Biotechnology Co, Limited, Shanghai, China) and a TECAN Infinite F50 enzyme-mark analyzer (Tecan, Männedorf, Switzerland). OIF levels were calculated based on the corresponding absorbance values of the standard curve generated using the ELISA kit OIF.

### 2.4 Statistical analysis

Data are expressed as mean value ± standard deviation. One-way analysis of variance (ANOVA) was applied to compare means between groups with normally distributed data. Data with a skewed distribution are presented as median ± interquartile range. Skewed data with a normal distribution after logarithmic transformation were analyzed using 1-way ANOVA. Skewed data were analyzed using a nonparametric test. Enumeration data were expressed by ratio and percentage with a chi-squared test used for comparison between groups. Correlations between serum level of OIF with UAER and eGFR were analyzed by Pearson correlation analysis. The predictive value of OIF for the risk of early-stage DN was assessed using multivariate logistic regression. Receiver operating characteristic (ROC) analysis was conducted to determine the cut-off value of OIF for predicting early-stage DN. All analyses were performed with Statistical Package for Social Sciences version 17.0 (SPSS, Chicago, IL). In all statistical tests, differences with *P* < .05 were considered significant.

## 3 Results

### 3.1 Characteristics of study subjects

The quartiles of serum OIF in the study population were quartile 1 (Q1) (>367.5 pg/mL), Q2 (320.3–367.5 pg/mL), Q3 (275.0–320.3 pg/mL), and Q4 (<275.0 pg/mL). The baseline clinical characteristics of the participants are shown in Table 1. The duration of diabetes, HCY, CYSC, laminin urinary albumin excretion rate (LnUAER), eGFR, and the prevalence of eGFR <90 mL/min and eGFR <60 mL/min were significantly lower in quartile 1 of serum OIF compared with the other quartiles. Other variables were not significantly different between quartiles of OIF.

**Table 1:**
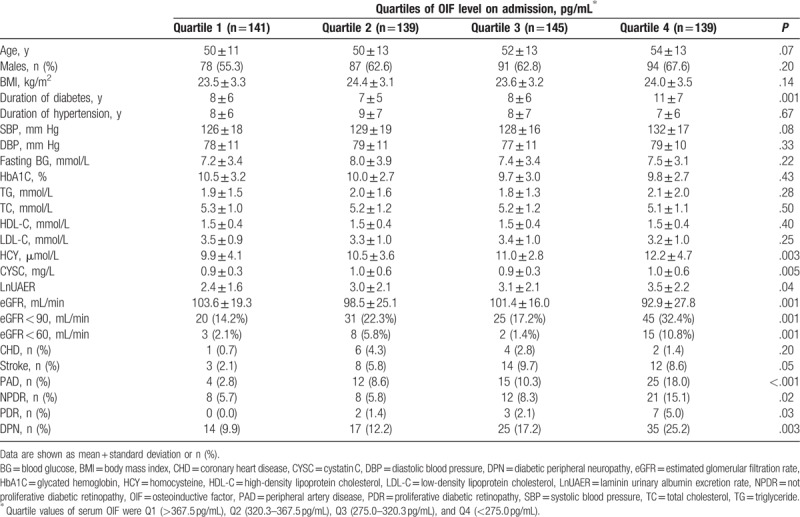
Baseline clinical characteristics.

Mean eGFR was lower in patients with DN (microalbuminuria or macroalbuminuria) than in those without DN (96.9 ± 17.3 mL/min, 54.1 ± 37.4 mL/min vs. 102.9 ± 16.6 mL/min; *P*_trend_ < .001). DN with microalbuminuria was associated with higher systolic blood pressure (SBP), higher occurrence of eGFR <90 mL/min, stroke, peripheral artery disease (PAD), not proliferative diabetic retinopathy (NPDR), proliferative diabetic retinopathy (PDR), and diabetic peripheral neuropathy (DPN) compared to patients without DN (*P* < .001). DN with macroalbuminuria was associated with higher levels of SBP, HCY, and CYSC, higher occurrence of eGFR <90 mL/min, eGFR <60 mL/min, CHD, NPDR, PDR, and DPN compared to patients with DN with microalbuminuria (*P* < .001) (Table 2).

**Table 2:**
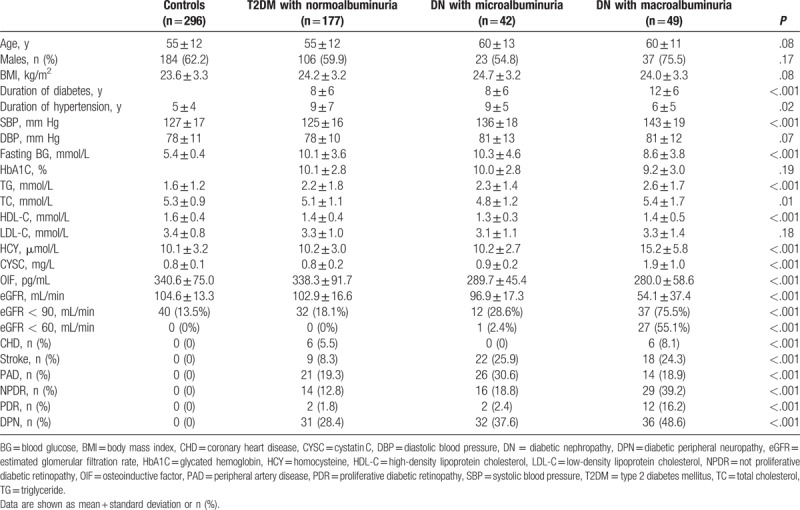
Baseline clinical characteristics and in-hospital clinical complications.

### 3.2 OIF levels were decreased in patients with DN, but not in T2DM

Serum OIF was reduced from 340.6 ± 75.0 pg/mL in healthy controls to 338.3 ± 91.7 pg/mL in patients with T2DM, and further reduced to 289.7 ± 45.4 pg/mL in DN patients with microalbuminuria, and 280.0 ± 58.6 pg/mL in DN patients with macroalbuminuria (*P*_trend_ < .001) (Table 2).

### 3.3 OIF levels were associated with proteinuria and complications resulting from T2DM

**Figure 1:**
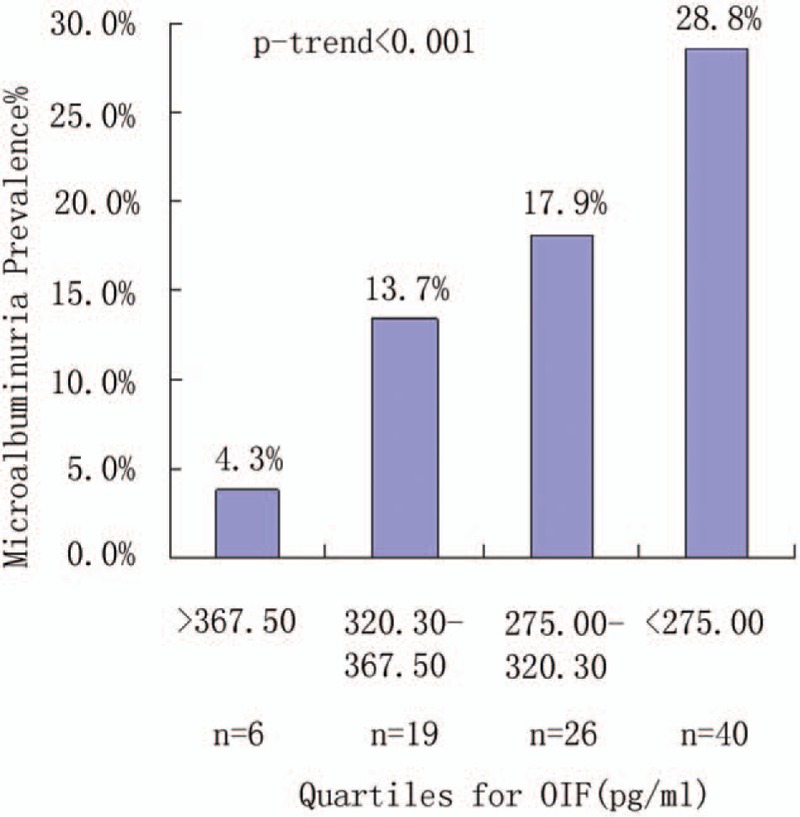
Prevalence of microalbuminuria by quartiles of serum OIF. The association between OIF and prevalence of microalbuminuria was significant (*P* < .001, overall and for trend). OIF indicates microalbuminuria. OIF = osteoinductive factor.

**Figure 2:**
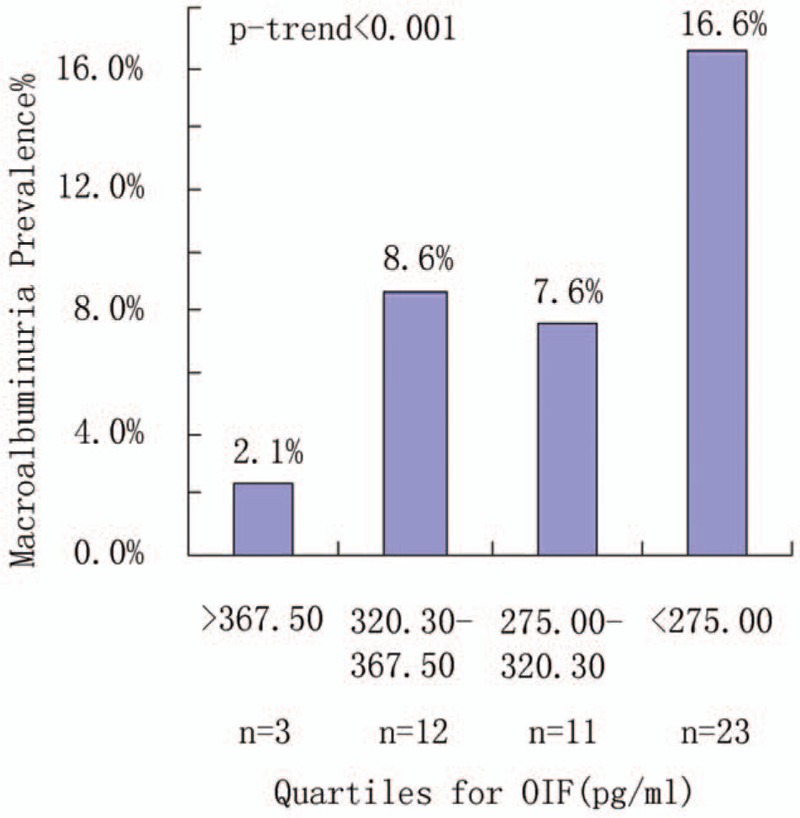
Prevalence of macroalbuminuria by quartiles of serum OIF. The association between OIF and prevalence of macroalbuminuria was significant (*P* < .001, overall). OIF = osteoinductive factor.

Univariate analysis showed that the prevalence of microalbuminuria was associated with serum OIF: with a prevalence of 4.3% for quartile Q1, 13.7% for Q2, 17.9% for Q3, and 28.8% for Q4 (*P*_trend_ < .001) (Fig. 1). Univariate analysis showed that the prevalence of macroalbuminuria was also associated with serum OIF: with a prevalence of 2.1% for quartile Q1, 8.6% for Q2, 7.6% for Q3, and 16.6% for Q4 (*P*_trend_ < .001) (Fig. 2). Patients with a lower serum OIF showed a higher rate of complications resulting from T2DM including stroke (2.1%, 5.8%, 9.7%, and 8.6% for Q1–Q4, respectively; *P* = .05), PAD (2.8%, 8.6%, 10.3%, and 18.0% for Q1–Q4, respectively; *P* < .001), NPDR (5.7%, 5.8%, 8.3%, and 15.1% for Q1–Q4, respectively; *P* = .02), PDR (0%, 1.4%, 2.1%, and 5.0% for Q1–Q4, respectively; *P* = .03), and DPN (9.9%, 12.2%, 17.2%, and 25.2% for Q1–Q4, respectively; *P* = .003 (Table 1).

Pearson correlation analysis showed that OIF was positively correlated with eGFR (*P* < .01), and negatively correlated with the duration of diabetes, SBP, HCY, CYSC, SCr, and LnUAER (*P* < .01) (Table 3).

**Table 3:**
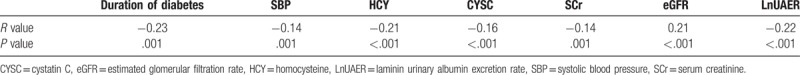
Pearson correlation analysis.

### 3.4 OIF may be a good predictor for early diabetic nephropathy

**Figure 3:**
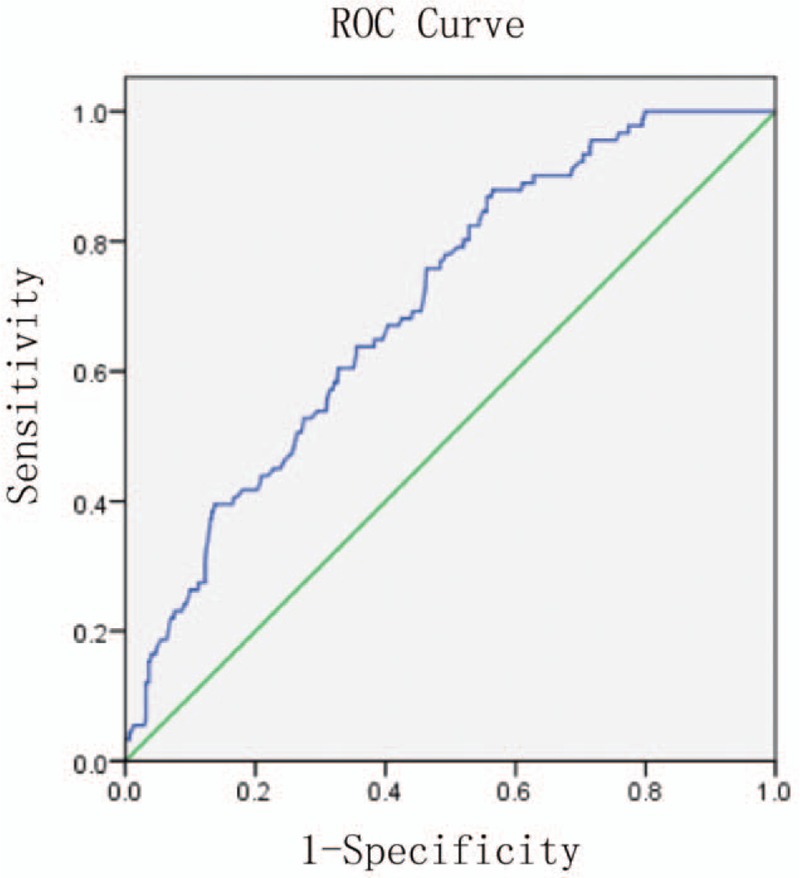
Receiver operator characteristic (ROC) curve analysis. ROC curve analysis showed that at a cutoff of 343.4, OIF exhibited 87.9% sensitivity and 56.4% specificity for detecting microalbuminuria (the area under the curve was 0.702). OIF = osteoinductive factor.

To investigate the potential use of OIF in the diagnosis of microalbuminuria in patients with diabetes we performed ROC curve analysis of OIF levels in T2DM patients. The area under the curve (AUC) was 0.702, and at a cutoff of 343.4 pg/mL, serum OIF exhibited a sensitivity of 87.9% and a specificity of 56.4% for detecting microalbuminuria (Fig. 3). Univariate logistic regression indicated that a serum OIF of <343.4 pg/mL was highly predictive of DN with microalbuminuria (odds ratio [OR] = 5.61; 95% confidence interval [95% CI], 2.91–10.81). Multivariate logistic regression performed including the variables OIF <343.4 pg/mL, eGFR <60 mL/min, eGFR <90 mL/min, FPG, TRIG, CHOL, HDL-C, LP(a), HCY, CYSC, CHD, stroke, PAD, NPDR, PDR, DPN, duration of diabetes, and hypertension showed that OIF <343.4 pg/mL was significantly associated with the occurrence of DN with microalbuminuria (OR = 11.60; 95% CI, 1.25–107.47) (Table 4).

**Table 4:**
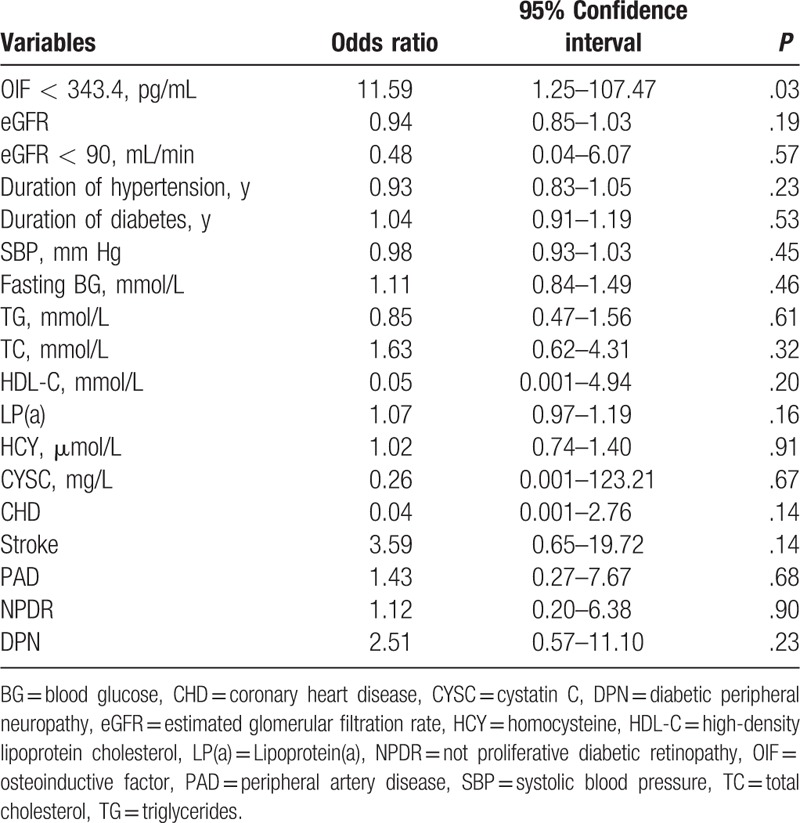
Multivariable association between diabetic nephropathy with microalbuminuria and osteoinductive factor.

In summary, OIF levels are decreased in patients with DN. Univariate analysis showed microalbuminuria prevalence negatively correlated with OIF, 4.3% for Q1 (>367.5 pg/mL), 13.7% for Q2 (320.3–367.5 pg/mL), 17.9% for Q3 (275.0–320.3 pg/mL), and 28.8% for Q4 (<275.0 pg/mL) (*P*_trend_ < .001), as did T2DM complications. ROC analysis showed an OIF of <343.4 pg/mL was predictive of DN (C statistic 0.702). OIF <343.4 pg/mL remained predictive of microalbuminuria (OR = 11.60; 95% CI: 1.25–107.47) after adjusting for confounding factors.

## 4 Discussion

We show that serum OIF is associated with the prevalence of DN with microalbuminuria in patients with T2DM. We also show that a serum OIF <343.4 pg/mL was an independent and significant predictor of DN with microalbuminuria. Moreover, a low serum OIF was strongly associated with T2DM complications. The positive correlation between serum OIF and eGFR, and its negative correlation with duration of diabetes, SBP, HCY, CYSC, SCr, and LnUAER, indicates that serum OIF reflects the development of DN in T2DM, similar to UAER and eGFR. This suggests that OIF levels are strongly associated with renal function and that the decline of serum OIF is closely related to the development and pathogenesis of DN. A reduction of OIF has been reported in the kidneys of diabetic mice indicating that OIF may be involved in the pathogenesis of diabetic fibrotic complications and the development of DN, which is consistent with our results.^[15]^ From nonparametric ROC plots, we found that serum OIF had a high sensitivity and specificity for the prediction of microalbuminuria (87.9% and 56.4%, respectively). The AUC of OIF for the prediction of microalbuminuria reached 0.702. This suggests that serum OIF has the potential to be a new predictive diagnostic marker of DN in patients with T2DM. However, Wang's study indicated that serum OIF was increased in patients with DN,^[6]^ which is contrary to our finding. The main reason for this difference may be that the duration of diabetes and diabetes nephropathy was different in the patients in the 2 studies; Wang did not specify the duration of DN in the patients, but in that study, eGFR in each group was higher than it was in the present study. We consider that OIF may be associated with GFR; OIF is high in the renal ultrafiltration state. With renal fibrosis and decreased GFR, OIF gradually decreases. However, this cannot explain why OIF increased in the macroalbuminuria group in Wang's study, and further research will be performed to explain this issue.

Hyperglycemia and hyperlipidemia are important factors in the development of DN. The long-term exposure of glomerular endothelial cells to high blood glucose and high TGs results in increased secretion of cytokines such as angiotensin II (AngII), transforming growth factor-β (TGF-β), and vascular endothelial growth factor. Cytokines will elevate TGF-β, and induce glomerular sclerosis and tubular interstitial fibrosis, which finally leads to DN.^[16,17]^ In vitro experiments suggest that cytokines related to vascular injury and angiogenesis including fibrocyte growth factor, TGF-β and AngII can reduce OIF expression.^[18]^ The activation of the AngII system is a major cause of renal hypertension supporting the finding of this research where SBP increased as OIF decreased with the deterioration of DN. Hyperlipidemia with increased LDL-C can promote the formation of foam cells in the vascular wall leading to atherosclerosis, thickening of the renal vascular wall and reduced elasticity, eventually resulting in DN.^[19,20]^ The expression of vascular OIF is reduced in atherosclerosis^[21–23]^ and reduced OIF expression induces proliferation of vascular smooth muscle cells (VSMC), increasing vascular endothelial injury and atherosclerosis plaque development.^[24]^ Decreased OIF together with nuclear transcription factor-κB (NF-κB) p65 signaling pathway activation correlated with DN pathogenesis in a diabetic mouse model. Reduced OIF may aggravate diabetic renal fibrosis partially by increasing the level of NF-κB p65.^[15]^ This suggests that OIF may play a direct role in DN though the development of renal fibrosis and atherosclerosis of the renal arteries; however, further research is necessary to understand the mechanisms involved.

Vascular calcification can be observed in chronic renal diseases, and metaplasia of VSMC to an osteoblastic phenotype linked to increasing expression of BMP-2 and BMP-3 is a feature of this vascular calcification in diabetes.^[25,26]^ The growth stimulating activity of OIF on bone acts through increased expression of BMP-2 and BMP-3, indicating that OIF would be expected to increase in DN. Various BMP have been identified in the kidney, including BMP-2, BMP-3, BMP-4, BMP-5, BMP-6, and BMP-7.^[27]^ BMP-7 is a member of the TGF-β superfamily,^[28]^ and is involved in renal protection by enhancing formation of normal renal structure and maintaining its normal physiological function, and also antagonizing TGF-β induced renal fibrosis by inhibiting the release of proinflammatory cytokines in renal proximal tubules and protecting glomerular podocytes, potentially delaying the development of DN.^[29]^ BMP-7 can also prevent metaplasia of VSMC to osteoblasts, thereby preventing vascular calcification.^[30]^ While greater serum OIF may increase vascular calcification and growth stimulating activity through BMP-2 and BMP-3, OIF may function to inhibit TGF-β, protecting glomerular podocytes and reducing renal vascular calcification through the actions of BMP-7. Conversely, reduced expression of OIF may result in renal interstitial fibrosis through the actions of TGF-β, AngII, and NF-κB p65, and atherosclerosis of renal arteries by TC, LDL-C, and LP(a), resulting in DN. Further studies are required to understand the complex interactions between OIF, TGF-β, NF-κB p65, and BMPs.

The small-scale cross-sectional nature of the present study, conducted at a single center and with a relatively small number of subjects, means that a longer follow-up will be required to determine the long-term predictive value of OIF. Further research is required to understand the mechanisms through which downregulation of OIF influences the risk of DN in patients with T2DM.

## 5 Conclusions

Reduced serum OIF is an independent diagnostic marker of DN with microalbuminuria in patients with T2DM. In addition, low serum OIF was associated with complications resulting from T2DM. OIF has the potential to be a new marker for screening patients at risk for early DN leading to increased prophylactic treatment for DN in these patients.

## Acknowledgments

The authors wish to thank the staff at the Department of Endocrinology, The First Hospital of Longyan Affiliated to Fujian Medical University for their generous help with data collection.

## Author contributions

**Data curation:** Rong Huang.

**Formal analysis:** Wen Wei.

**Methodology:** Rong Huang.

**Project administration:** Mei Tu.

**Supervision:** Mei Tu.

**Validation:** Tong Chen.

**Writing – original draft:** Wen Wei.

**Writing – review and editing:** Mei Tu.
